# Beyond Ebola treatment units: severe infection temporary treatment units as an essential element of Ebola case management during an outbreak

**DOI:** 10.1186/s12879-017-2235-x

**Published:** 2017-02-06

**Authors:** Christian Janke, Katrin Moira Heim, Florian Steiner, Moses Massaquoi, Miatta Zenabu Gbanya, Claudia Frey, Guenter Froeschl

**Affiliations:** 1 NATO Centre of Excellence for Military Medicine, Deployment Health Surveillance Capability, Dachauer Str. 128, 80637 Munich, Germany; 2 German Red Cross, Berlin, Germany; 3 Medical Department, Division of Infectiology and Pneumonology, Charité - Universitätsmedizin, Charitéplatz 1, 10117 Berlin, Germany; 4 Ministry of Health and Social Welfare of Liberia/Incidence Management System of Liberia, Ebola Case Management Team, Monrovia, Liberia; 5 Department of Tropical Medicine at the Bernhard Nocht Institute, German Armed Forces Hospital Hamburg, Bernhard-Nocht-Straße 74, 20259 Hamburg, Germany; 6 Division of Infectious Diseases and Tropical Medicine, Medical Centre of the University of Munich, Leopoldstr. 5, 80802 Munich, Germany; 7 German Centre for Infection Research (DZIF), Partner Site, Munich, Germany

**Keywords:** Ebola, Liberia, Case management, Ebola treatment unit, Severe infections teamporary treatment unit, SITTU, Infectious disease unit, Isolation, Epidemiology, Nosocomial infection

## Abstract

In the course of the Ebola outbreak in West Africa that was witnessed since early 2014, the response mechanisms showed deficits in terms of timeliness, volume and adequacy. The authors were deployed in the Ebola campaign in the West African country Liberia, where by September 2014 the changing epidemiological pattern made reconsiderations of guidelines and adopted procedures necessary. A temporary facility set up as a conventional Ebola Treatment Unit in the Liberian capital Monrovia was re-dedicated into a Severe Infections Temporary Treatment Unit. This facility allowed for stratification based on the nosocomial risk of exposure to Ebola virus for a growing subgroup of admitted patients that in the end would turn out as Ebola negative cases. At the same time, adequate diagnostic measures and treatment for the non-Ebola conditions of these patients could be provided without compromising work safety of the employed staff. The key elements of the new unit comprised a Suspect Cases Area similar to that of conventional Ebola treatment units for newly arriving patients, an Unlikely Cases Area for patients with a first negative Ebola PCR result, and a Confirmed Negative Cases Area for patients in whom Ebola could be ruled out. The authors, comprising representatives of the Liberian Ministry of Health and Social Welfare, as well as infectious disease specialists from the German Ebola Task Force are presenting key features of the adapted concept, and are highlighting its relevance in raising acceptance for outbreak counter-measures within the population at stake.

## Background

With the aim of reducing the risk of nosocomial Ebola infections within the facility [1], separating patients according to their individual probability of having Ebola Virus Disease (EVD) into three distinct categories (Suspect, Probable and Confirmed Positive Cases) is a core part of an Ebola Treatment Unit (ETU). However, during most of the EVD outbreak in West Africa in 2014 to 2016, the suspected cases were not classified further.

Although an ETU is considered a suitable and pragmatic setting for managing Ebola patients, it is less effective for managing the needs of non-Ebola patients due to the risk of nosocomial infection and lacking availability of diagnostic tools and differentiated therapeutic regimens for non-Ebola conditions. Patients with Ebola-like symptoms, however, were often denied access to public health care facilities during the outbreak, leaving them entirely without an adequate health care option. According to the personal experience of the authors in the course of the Ebola campaign and patient feedback gathered in informal interviews, some patients with Ebola-like symptoms preferred to stay at home rather than to attend an ETU, owing to the possible risk of becoming infected with the Ebola virus. Furthermore, ETUs were seen as a facility with a limited level of care and a high mortality rate of well over 50%. A patient who was admitted to an ETU and who later proved to be negative for EVD may potentially have been exposed to EVD within the facility and thus remained an “Ebola contact” by definition [2]. In addition, in the ETU the patient’s therapy was limited to a rudimentary spectrum of empirical treatments for all conditions other than Ebola. Following discharge from the ETU, these contact cases had little realistic chance of obtaining subsequent inpatient treatment at an ordinary health care facility in the setting of the West African EVD outbreak [3].

Over the course of an Ebola outbreak, the positive predictive value of a given case definition varies significantly due to changes in Ebola prevalence rates. In September 2014 when the Ebola prevalence was at its highest in Liberia, nine out of ten suspect cases tested positive for the Ebola virus [4]. Towards the end of 2014 this situation changed substantially. Fewer patients admitted to ETUs eventually tested positive for the Ebola virus, reflecting the considerably decreased positive predictive value of the Ebola case definition in a low-incidence phase of the outbreak. At that time around nine out of ten suspected cases in any Liberian ETU turned out to be Ebola virus negative and were thus unnecessarily exposed to substandard care for conditions other than Ebola and possibly also to still Ebola-infected patients in the suspect wards. The altered balance between benefits and risks by presenting to an ETU with a febrile condition resembling EVD were also recognized by the patients. It is likely that this situation has had a crucial impact on health decision making in the general public at that time. Decreasing overall patient numbers presenting themselves at the screening facilities corroborate this possibility, although EVD-like illnesses such as malaria should have continued to produce case numbers that should have been comparable to peak periods of the Ebola outbreak. During all phases there should have been numerous individuals fulfilling the EVD case definition; at peak times patients actually carrying Ebola virus, and in later phases patients that were less frequently true Ebola cases and more frequently cases of just Ebola-like diseases.

This was the situation in Liberia when on December 23rd, 2014 the SKD2 ETU in Monrovia, named after the Samuel Kanyon Doe Stadium nearby became operational. The large size of the SKD2 facility and increased facility quality requirements commissioned by national and inter-governmental stakeholders proved to be a challenge for the speed at which it became operational. Coupled with setbacks during the construction period due to delayed delivery of building materials (e.g., high temperature incinerator) and delivery of broken materials (e.g., sewer pipes), the construction time for the installation was about 4 months.

This ETU was designed as a WHO standard 100 bed ETU and was supported by the German government with deployment of material and staff from both the German Red Cross and the German Armed Forces. By the end of December 2014 there were more than 1000 ETU beds available countrywide while the number of hospitalized suspected Ebola cases had fallen to below 100 in the entire country. Due to the remaining fear of Ebola many patients with non-EVD infectious diseases were not admitted by ordinary health care facilities as they were not prepared to manage potentially highly contagious patients [5].

When the Ebola crisis was at its peak there may not have been sufficient resources available to address this problem. However, by December 2014 adequate resources had been deployed in West Africa to enable consideration of how best to manage non-EVD patients with severe infectious diseases. The assumption that an ETU is ethically, epidemiologically and medically an appropriate place to admit any patient with Ebola-like signs and symptoms was no longer sustainable.

## Methods

Driven by these considerations and guided by a request of the Incidence Management System of the Liberian Ministry of Health (MoH/IMS) to implement an adapted case management concept the German Red Cross and German Armed Forces joined efforts and expertise to meet this task. There was a substantial need to conceive and materialize a facility meeting the current epidemiological situation at that time, which would be suitable for diagnosis and treatment of other relevant infectious diseases in the context of an ongoing Ebola outbreak. The pilot project of a Severe Infection Temporary Treatment Unit (SITTU) was launched. In conventional ETUs, patients were separated according to their risk of being infected by Ebola virus. Whereas in the SITTU design patients were separated by their probability of not being infected by Ebola virus into suspect cases (any patient presenting and fulfilling case definition criteria), unlikely cases (patients with one negative Ebola Polymerase Chain Reaction (PCR) result after admission) and confirmed negative cases (patients testing negative for EVD twice 48 h apart).

**Fig. 1:**
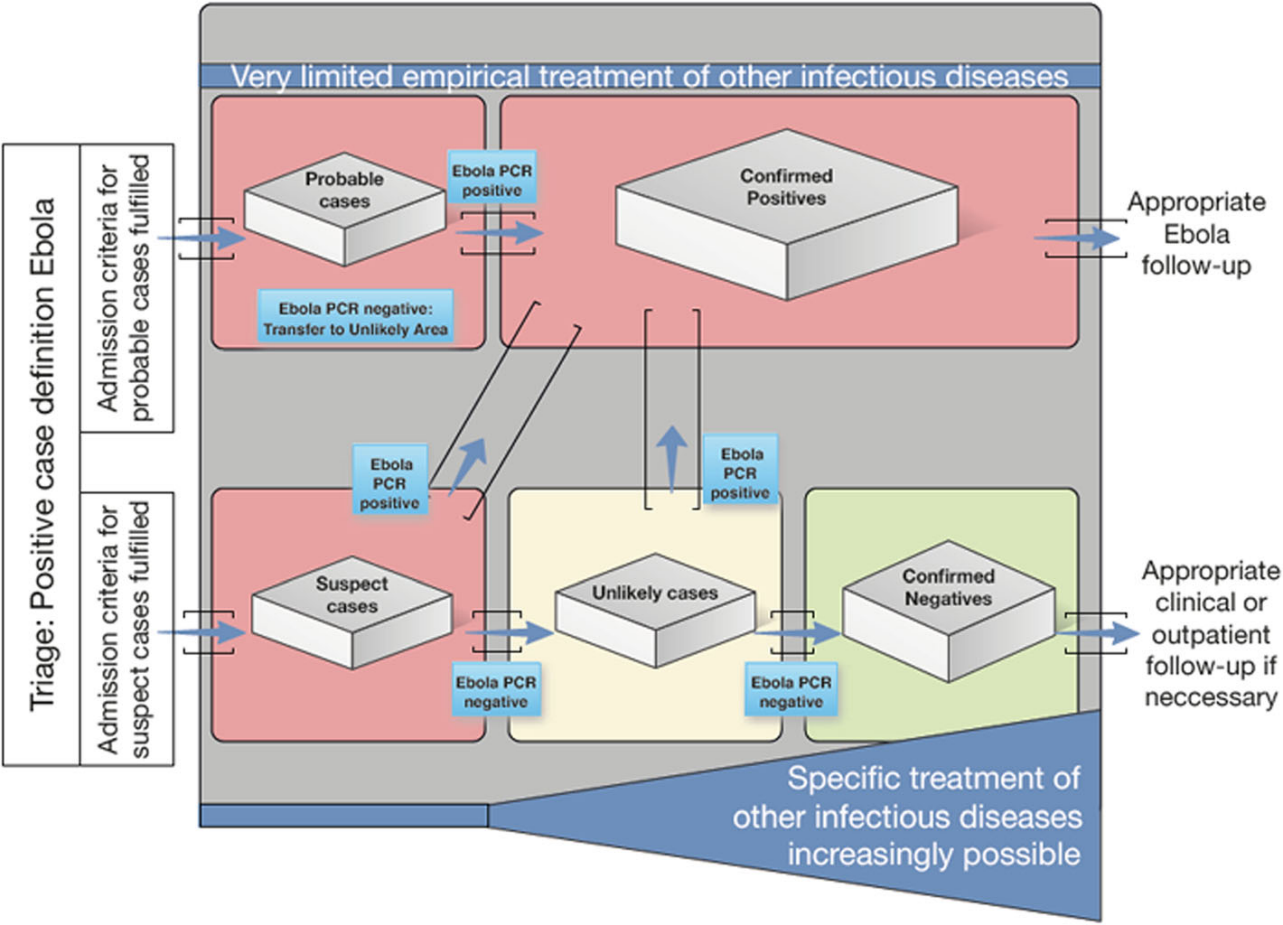
The concept of an optimized ETU. The Severe Infection Temporary Treatment Unit (SITTU) in Monrovia consisted of a Suspect Cases Area, an Unlikely Cases Area and a Confirmed Negative Cases Area, thereby representing only a part of the idealised facility pictured above (*lower half* of the image). In the Monrovia scenario the complementary parts (as shown in the *upper half* of the Fig. 1) were represented by pre-existing conventional ETUs near the SITTU. The two additional areas (Unlikely Cases Area and Confirmed Negative Cases Area, in the lower half of the figure) allow an allocation of patients according to their risk of not being infected by Ebola virus with the aim of reducing the risk of nosocomial Ebola virus infections and facilitating an adapted medical care

**Fig. 2:**
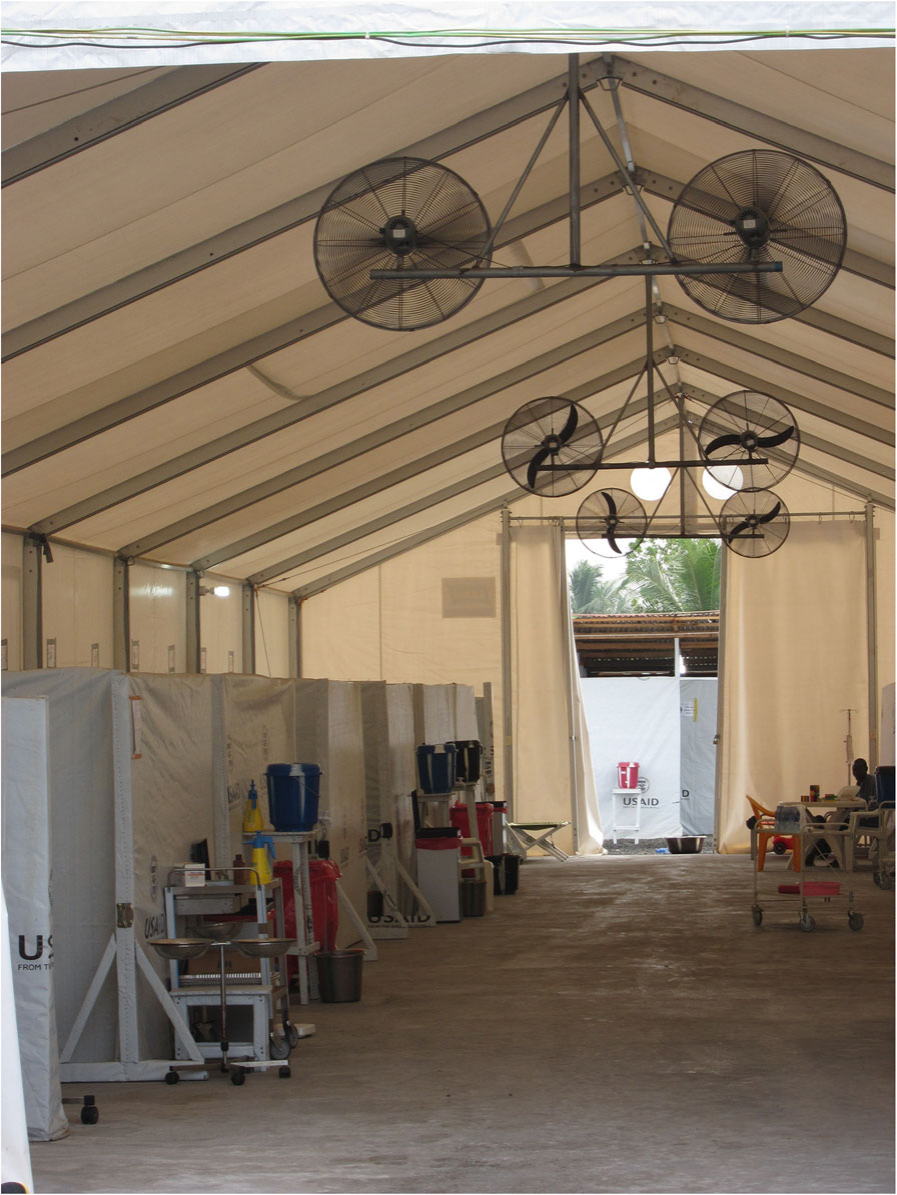
Interior View of the SITTU. The aluminium and sheeting structure is built on sealed concrete foundations. In the cubicles on the left, each compartment is dedicated to one individual patient. Within these cubicles only strictly personalized items such as chairs, drip stands and closets are placed. Individual buckets were provided for personal hygiene and sanitation to avoid mixing of the patients in lavatories or toilets. The patients were requested not to move within the structure

Accompanied by a conventional ETU, either within the facility complex or in close proximity, and by health care facilities of the recovering national health services, the SITTU design constitutes an integrated and optimized patient management and treatment system (Fig. 1). In a SITTU, just as in a conventional ETU, the patient flow is unidirectional, starting from Suspect Cases Area to the Unlikely Cases Area, to eventually the Confirmed Negative Cases Area, as patients are segregated along the risk of not having EVD. Patients fulfilling the case definition criteria are admitted from the triage area to the Suspect Cases Area for a very short period of time until the first Ebola PCR result is available. The length of time for this initial assessment depends on the availability of quick PCR-results or alternative rapid tests which may be available in the future. The design of this area is restrictive in terms of inter-patient contact and processes are designed to minimize the risk of a nosocomial Ebola infection while the test is pending. Patient areas within the Suspect Cases Area in the SITTU are designed as spacious cubicles, allowing for a more stringent isolation of patients, while simultaneously providing a greater degree of individual privacy (Fig. 2).

**Fig. 3:**
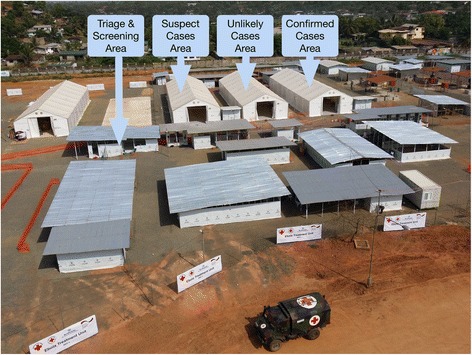
The German Severe Infections Temporary Treatment Unit. The German Unit at the SKD Football Stadium in Monrovia, Liberia, previously designed as a regular Ebola Treatment Unit, now re-designed into a Severe Infection Temporary Treatment Unit, representing the lower half of Fig. 1. In the course of increasing evidence of non-Ebola virus infection patients are referred between wards from left to right. In case of a positive Ebola virus PCR result patients are referred to a conventional ETU nearby. Please note that the banners at the fence are still presenting an ETU; these banners date back to the start of construction of the facility, when it was still meant to be an ETU. After rededication into a SITTU the banners remained unchanged

Immediately following receipt of the first negative Ebola result, patients are transferred to the Unlikely Cases Area (Fig. 3) where a 2nd PCR is performed after 48 h. At this stage in the diagnostic algorithm, while the Ebola Virus Disease cannot be entirely excluded, a potentially infected patient is highly unlikely to be infectious to other patients due to the low viral load which may have rendered the first test result negative in an assumedly early phase of infection. The rationale for the 48-h time interval was adopted from the Liberia Ebola Virus Disease Clinical Management Manual issued by the MoH/IMS [6]. In early phases of an acute Ebola virus infection viral loads may still be found below the limit of detection of any of the PCR assays that were in use at that time. However, within 48 h the large majority of these early cases is expected to have developed sufficiently high viral loads to become detectable.

The key consideration of the Suspect Cases Area is to maximise patient and staff safety, as here the eventual infection status of each individual patient remains incalculable. Whereas the Unlikely Cases Area is primarily a holding area for patients where a critical contagiousness for Ebola virus can be virtually ruled out, which, in turn, translates into a reduced risk for potential cross-contamination. However, staff safety still remains crucial, requiring personal protective measures until patients test negative for the Ebola virus a second time. Any positive Ebola PCR result either in the Suspect Cases Area or in the Unlikely Cases Area would lead to the immediate referral of the corresponding patient to a conventional ETU nearby.

When the second negative Ebola PCR taken 48 h after admission is established, patients are either released from the facility if no further clinical management is necessary, or they are transferred from the Unlikely Cases Area to the Confirmed Negative Cases Area for further inpatient care. Both the release from the facility and the referral to the Confirmed Negative Cases Area require an individual decontamination procedure of the patient, involving the discarding of all clothing and personal items and a thorough full body shower with 0.05% chlorinated water under observation of a staff member of equal sex. In the Confirmed Negative Cases Area the clinical management changes significantly. The Personal Protective Equipment (PPE) used by the medical staff is reduced to a conventional infectious disease unit standard. Consequently, the acceptable working time and the ability to perform diagnostic procedures as well as invasive medical interventions are extended substantially. A field laboratory for blood count and clinical chemistry, a variety of rapid tests, urine analysis, microscopy and a mobile ultrasound device facilitate the shift from empirical treatment to diagnosis-based therapies. Confirmed negative patients can also be sent to external X-Ray examinations or referred to specialized departments such as obstetrics.

In an environment that remained governed by fear of Ebola, the general collapse of health services and the drain of health professionals to Ebola Treatment Units meant that transferring patients to Liberian hospitals proved to be extremely challenging. The SITTU was able to build up close working relations with local health care facilities. This was important as it enabled the SITTU to reintegrate patients into the national health care system. This included patients with chronic infectious diseases such as HIV/AIDS and Tuberculosis as well as patients with an acute need of surgical interventions.

When the SITTU was finally operational and opened, patients were admitted from different sources. Due to the breakdown of the national health care system some patients were transferred directly from conventional ETUs to the SITTU after they had received one or two negative Ebola PCR results, if they still were in need of continued inpatient care. Numerous patients fulfilling the Ebola case definition were brought in from the community by the Community Health Teams or presented in the triage area voluntarily.

## Results

**Fig. 4:**
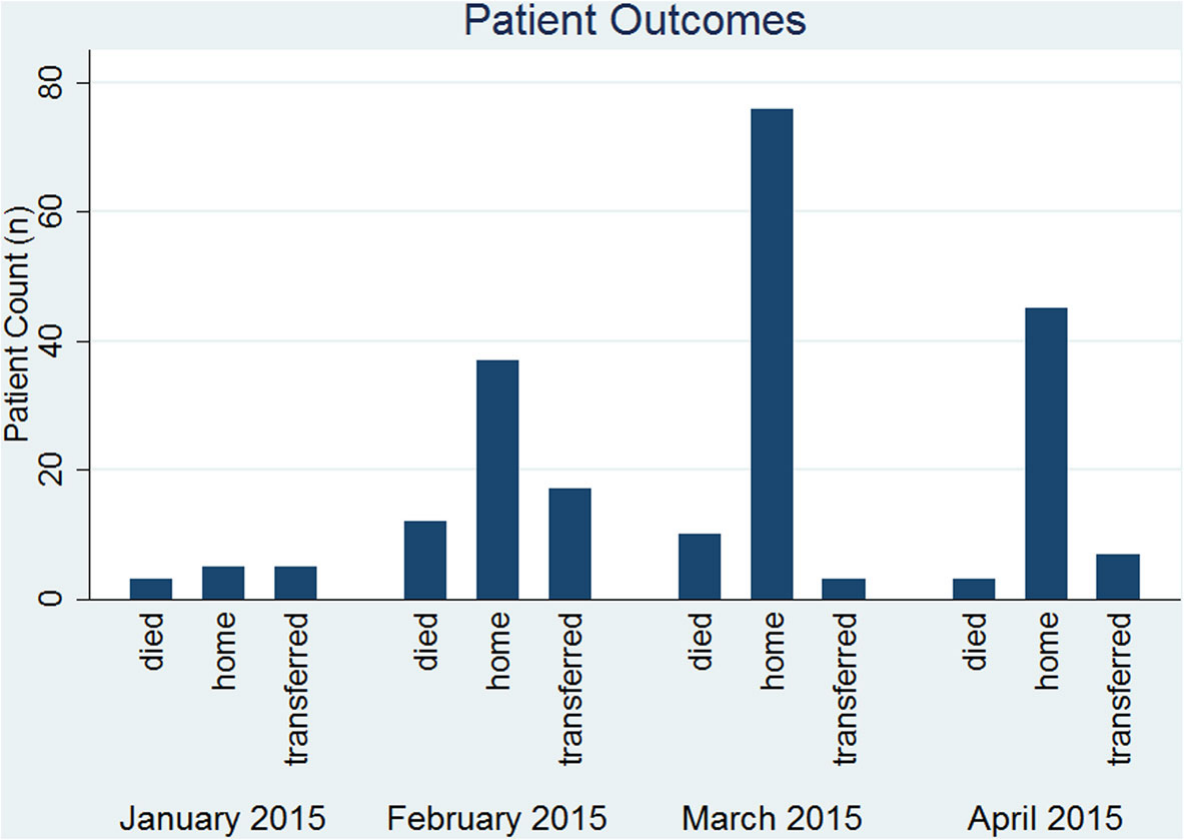
Patient Outcome at the SITTU. Outcome variable for patients is categorized as “died” for all patients that were admitted alive and died within the SITTU, “home” for all patients that could be released into their community, requiring none or minimal further medical care, and “transferred” for all patients that were transferred to other health care facilities for specialized care

In total 536 patients were registered during triage at the SITTU in the course of its operation between January and April 2015. Of the 536 documented patients that were screened in the triage area, 224 met case definition criteria and were admitted to the SITTU. Eight patients left against medical advice and were followed up by our psychosocial unit. The SITTU closed on April 19th. In total 28 patients died in the facility, and 27 patients were referred to other health care facilities for further treatment. In three patients the outcome was undocumented. The remaining 158 patients recovered after treatment within the Confirmed Negative Cases Area and were eventually discharged (Fig. 4).

**Fig. 5:**
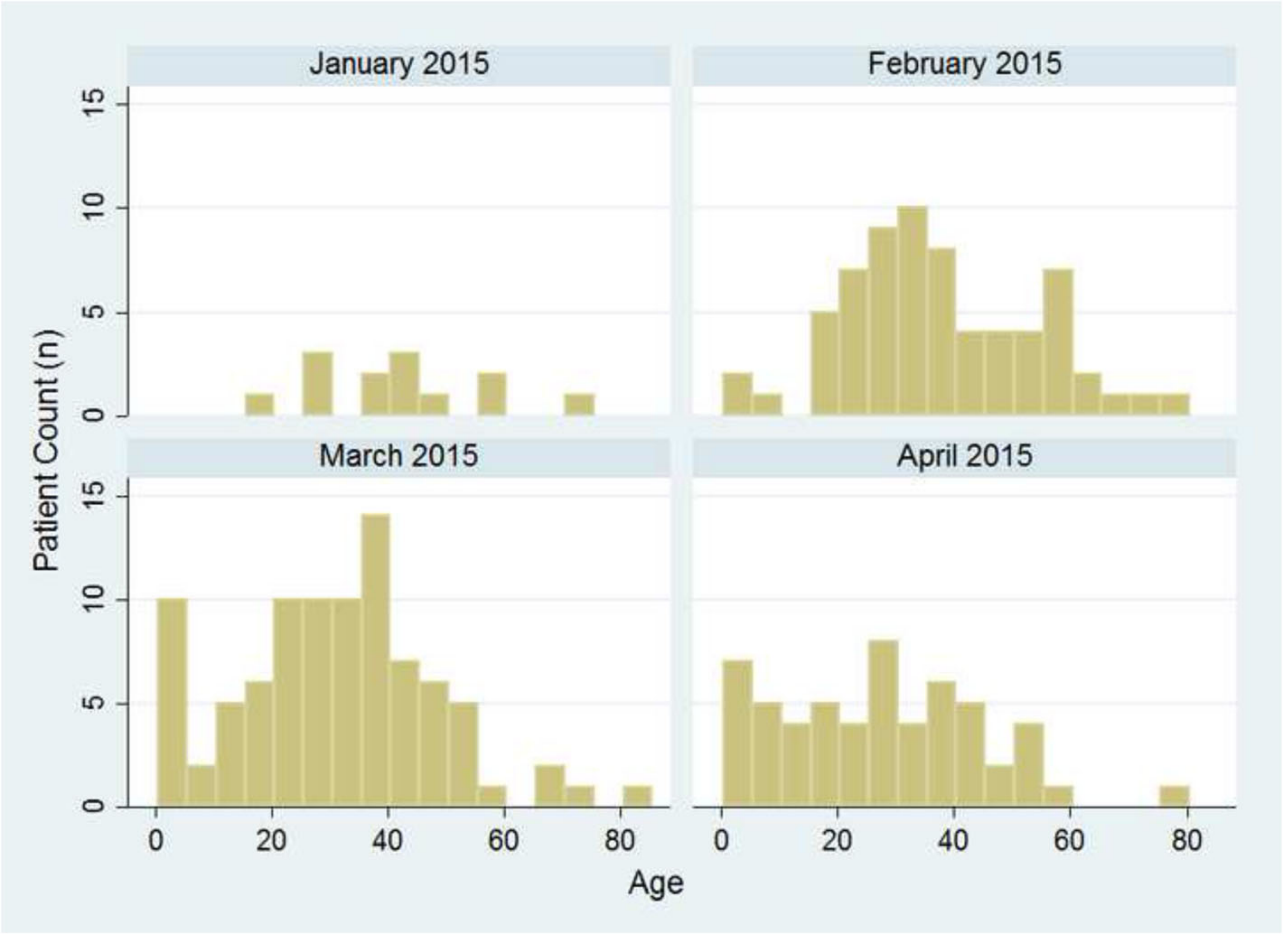
Age Distribution of Admitted SITTU Patients. The histogram with a bin width of 5 years shows the age distribution for each operational calendar month

The SITTU primarily treated adults and only a few children (Fig. 5). In Monrovia a facility by Doctors Without Borders (MSF) admitted children under the age of 5 years with Ebola-like symptoms for further diagnostic testing and treatment. Therefore, this patient group was mostly referred directly to the MSF facility, leaving this age group underrepresented at the SKD2 facility. The remaining admitted children under the age of 5 years and all pregnant women were placed in the maternity ward of SKD2, an additional annex consisting of a small ward where patients were located all along the diagnostic cascade, until they were either referred to an external ETU if tested positive or transferred to the Confirmed Negative Cases Area if tested twice negative for Ebola virus. Overall, 56.4% of the patients admitted to the SITTU were male, and 43.6% female.

As far as could be determined, the patients admitted had not had any contact with a known Ebola case or body fluids of severely ill index patients suspected of being EVD cases. Only two patients had attended a funeral in the past month. Unfortunately, the data on risk profile of admitted patients are incomplete because some patients were too ill to partake in a complete interview. Only three patients stated to have worked as health care workers.

The majority of admitted patients were eventually diagnosed with malaria, followed by gastroenteritis and HIV/AIDS-associated complications and acute respiratory infections (Table 1). In April 2015 an increase in patients with measles was detected with two admitted cases. Comparable to the local availability of diagnostic means prior to the Ebola outbreak many diagnoses in the SITTU were still based on clinical presentations alone, such as in cases of suspected measles.

**Table 1:** Morbidity Profile of Admitted SITTU Patients

	Malaria	Gastro-enteritis	HIV/AIDS	ARI	Other	Total
January	2 (16.7%)	0 (0%)	1 (8.3%)	1 (8.3%)	8 (66.7%)	12 (100%)
February	13 (19.7%)	7 (10.6%)	5 (7.6%)	8 (12.1%)	33 (50.0%)	66 (100%)
March	28 (31.1%)	6 (6.7%)	13 (14.4%)	5 (5.6%)	38 (42.2%)	90 (100%)
April	32 (57.1%)	8 (14.3%)	2 (3.6%)	2 (3.6%)	12 (21.4%)	56 (100%)
Total	75 (33.5%)	21 (9.4%)	21 (9.4%)	16 (7.1%)	91 (40.6%)	224 (100%)

Most frequent diagnoses, by month of admission (% of all patients admitted in the corresponding month). *ARI* acute respiratory infections (upper and lower combined)

**Fig. 6:**
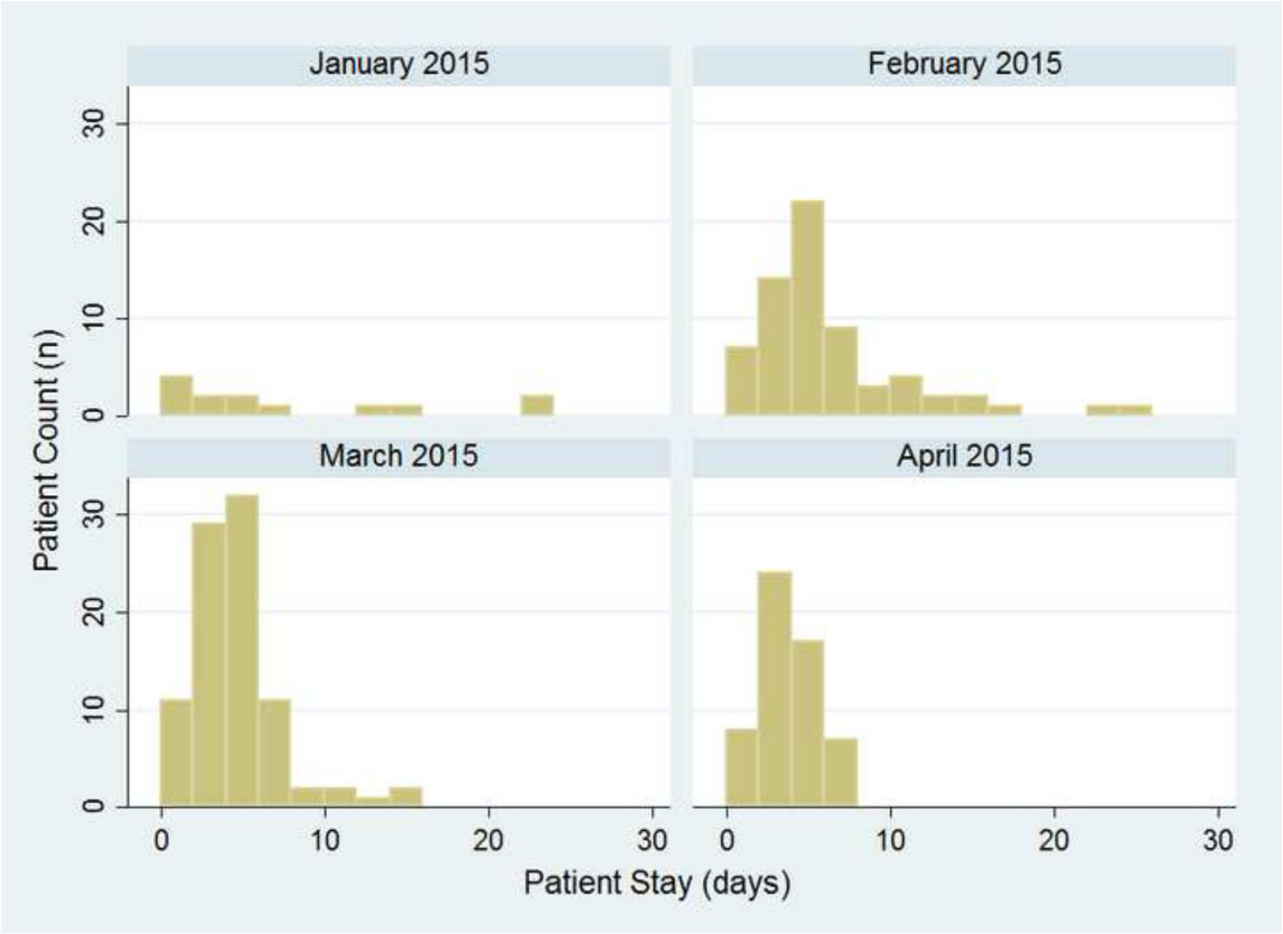
Length of Admission over Time at the SITTU. The histogram with a bin width of 2 days shows the patient counts on the y-axis over length of admission time on the x-axis, for each operational calendar month

The aim of the SITTU was to treat and isolate patients with Ebola-like symptoms and discharge or transfer them to the Confirmed Negative Cases Area once they tested negative for Ebola twice over the course of 48 h. In the initial phase the transfer options from the SITTU to other specialized facilities within the capital Monrovia for confirmed EVD negative patients were practically nonexistent. Due to reopening clinics, national health care projects and improved networking with additional health care facilities the patients’ holding time within the SITTU decreased over time (Fig. 6).

No patient admitted to the SITTU tested positive for EVD. Confirmed patient numbers across Liberia dropped to single case levels soon after the SITTU opened. Lacking an ongoing reason to be, the SITTU was closed after the last reported Liberian Ebola case at the end of April 2015. Liberia was declared free of Ebola virus transmission for the first time on May 9th 2015.

## Discussion

The SITTU is an entirely new concept and can be applied and adapted to many different settings. In the 2014 to 2016 Ebola outbreak it filled the gap between the existing health care facilities and the ETUs. During this time, it is estimated that due to the collapse of the health care system Ebola could have caused more than 10,000 additional deaths from malaria alone [7]. The question is raised as to how far an Unlikely Cases Area and even a Confirmed Negative Cases Area can still be an integral part of Ebola case management. Although these components admittedly focus on non-Ebola cases, we contend that they do contribute an added value to the overall Ebola case management system in three ways. First and foremost, an improved case management realized through the SITTU concept makes exposure of non-Ebola patients to EVD patients within the treatment facility extremely unlikely. Second, the SITTU provides a substantially higher level of clinical care for non-Ebola patients, thereby improving their outcome as well as potentially increasing acceptance of Ebola-specific facilities by patients with Ebola-like symptoms. The SITTU plays an essential part in identifying the remaining few index patients. From an epidemiological point of view, the last point is of paramount importance for ensuring that the transmission cycle is broken through sufficient numbers of true EVD cases being isolated. Third, ordinary health care facilities are protected from potential Ebola patients that they are ill-equipped to manage.

During the pilot project in Monrovia the SITTU approach proved to be effective beyond a theoretical concept. The concept was developed in Liberia at the end of 2014 for a number of reasons: in order to meet emerging necessities; so as to support an understanding within the community that continued screening efforts are crucial for identifying the final cases in the Ebola response; as well as to allow for a controlled and safe re-installation of the regular national health services, and to provide adequate health services to the large majority of patients showing Ebola-like symptoms yet suffering from other infectious diseases.

The authors are aware and critical of the substantial delay that the facility they were responsible for in Monrovia suffered from until finally becoming operational. However, in the course of the rather short operationalization of the SITTU it became very clear that this concept was an essential contribution to patient care in the midst of an EVD outbreak. It is hoped that with the dissemination of the hereby presented experiences a future implementation of the SITTU concept in the course of an outbreak of a highly infectious disease will arrive in a timely and integrated manner.

In conclusion, the SITTU concept provides a bridge between the emergency response phase, as represented by ETUs, and the recovery phase of national health services. This transitional period also allows staff to be reassured, equipment reallocated and guidelines on safe structures and procedures readjusted. Ebola Treatment Units are exceptionally complex from an ethical, medical and epidemiological point of view. During an outbreak each of these aspects has to be considered separately and continuously so as to adjust treatment policies in response to changing epidemiological patterns and behavioral dynamics. The Severe Infection Temporary Treatment Unit was a necessary and useful adaptation of Ebola case management protocols during the Ebola outbreak in Liberia, and may be transferrable to other settings.

## Abbreviations

ETU: Ebola treatment unit
EVD: Ebola virus disease
MoH/IMS: Ministry of Health/Incidence Management System
MSF: Médecins sans Frontières/Doctors without Borders
PCR: Polymerase chain reaction
PPE: Personal protective equipment
SITTU: Severe infections temporary treatment unit
SKD: Samuel Kanyon Doe (name of a sports compound including a large football stadium in Monrovia)
